# Immersive Technologies for Cognitive Rehabilitation in Dementia and Mild Cognitive Impairment: Systematic Review

**DOI:** 10.2196/84349

**Published:** 2026-03-18

**Authors:** Jaromir Konecny, Giuseppe Lanza, Serafino Buono, Raffaele Ferri, Antonina Luca, Radek Martinek, Sabrina Musso, Aurora Palmigiano, Barbora Prauzkova, Angelica Quercia, Francesco Rundo, Ramachandran Avala Subramanian, Alessandro Serretti, Michal Prauzek

**Affiliations:** 1 Department of Cybernetics and Biomedical Engineering, VSB – Technical University of Ostrava, 17. listopadu 2172/15, Ostrava - Poruba, 708 00, Czech Republic, +420 596 995 857; 2 Department of Surgery and Medical-Surgical Specialties, University of Catania, Catania, Italy; 3 Department of Clinical Neurosciences, Oasi Research Institute-IRCCS, Troina, Italy; 4 Department of Medicine and Surgery, “Kore” University of Enna, Enna, Italy; 5 Department of Management, VSB – Technical University of Ostrava, Ostrava - Poruba, Czech Republic; 6 Unit of Bioinformatics and Statistics, Oasi Research Institute-IRCCS, Troina, Italy

**Keywords:** cognitive rehabilitation, dementia, CAVE system, virtual reality, augmented reality, mild cognitive impairment, mixed reality, PRISMA, Cave Automatic Virtual Environment system

## Abstract

**Background:**

Cognitive decline across the mild cognitive impairment (MCI)–dementia continuum is a major driver of loss of independence and growing health- and social-care burden. Immersive technologies, such as virtual reality (VR), augmented reality (AR), and Cave Automatic Virtual Environment (CAVE) systems, are increasingly explored as tools to enhance engagement, personalization, and ecological validity in cognitive rehabilitation.

**Objective:**

This systematic review synthesizes current evidence on the usability, therapeutic effects, and implementation challenges of immersive technologies for cognitive rehabilitation in MCI and dementia.

**Methods:**

A systematic search of Scopus and Web of Science was conducted for peer-reviewed journal articles published between 2021 and 2026. Eligible studies investigated VR, AR, or CAVE interventions targeting cognitive rehabilitation outcomes in MCI and/or dementia and reported measures related to usability or acceptability, or cognitive, functional, or behavioral outcomes. Due to heterogeneity in technologies, intervention content, and outcome measures, findings were synthesized narratively with comparisons across modalities and study designs.

**Results:**

In total, 119 studies met the inclusion criteria. Across immersive VR interventions, signals of benefit were most consistently reported for memory, attention, and executive functioning, with several studies also targeting outcomes with higher ecological relevance (eg, everyday task performance and functional skills). AR approaches primarily support context-aware cueing and task guidance in real-world settings, aiming to strengthen daily functioning and independence. CAVE-based systems were frequently used for spatial navigation and embodied interaction, offering advantages for supervised clinical deployment. Key barriers included cybersickness and comfort issues, interface complexity, and onboarding demands in cognitively impaired users, limited accessibility and standardization of outcome measures, small samples and short follow-up periods, and practical constraints related to cost, space, staffing, and caregiver involvement.

**Conclusions:**

Immersive VR, AR, and CAVE systems are feasible and often engaging for cognitive rehabilitation in MCI and dementia, with promising therapeutic signals but substantial uncertainty driven by methodological and implementation heterogeneity. Future work should prioritize standardized reporting (intervention components, dose, and adverse events), clinically meaningful outcomes (including functional end points), adequately powered comparative trials, and explicit evaluation of scalability and real-world deployment pathways.

## Introduction

Dementia represents a rapidly growing global health challenge. In 2019, approximately 55 million people were estimated to be living with dementia worldwide, and this number is projected to rise to 139 million by 2050, underscoring the urgency of scalable, evidence-based rehabilitation and support strategies [1]. Cognitive decline across the mild cognitive impairment (MCI)–dementia continuum is a leading driver of loss of independence in later life and a growing burden for health and social care systems. MCI is commonly conceptualized as an intermediate clinical state between typical cognitive aging and dementia, but its course is heterogeneous—some individuals remain stable or improve, whereas others progress to dementia—which makes early, targeted intervention both attractive and complex [2-4]. Dementia syndromes, including Alzheimer disease (AD), involve progressive impairments across multiple cognitive domains with downstream effects on everyday functioning and quality of life, reinforcing the need for interventions that are not only clinically meaningful but also feasible in real-world settings [5,6].

Nonpharmacological approaches remain central to cognitive rehabilitation and support, particularly because disease-modifying pharmacotherapies are limited in scope and may not address day-to-day functional challenges across disease stages [5,7]. Conventional cognitive training and rehabilitation can improve specific skills, yet long-term engagement, personalization to heterogeneous deficits, and transfer to everyday functioning are persistent challenges [5,7,8]. In this context, outcomes related to activities of daily living (ADL) and instrumental activities of daily living (IADL) are especially important because they capture functionally meaningful abilities that shape independence and care needs [9]. Digital and computer-based interventions have therefore been explored as scalable tools for cognitive support, but questions remain regarding ecological validity, long-term adherence, and implementation barriers outside controlled research contexts [7,8].

Immersive technologies are increasingly proposed as a route to address these limitations by enabling interactive, multisensory, context-rich tasks that can approximate real-life demands. Conceptually, these systems can be situated within the broader extended reality (XR) landscape and the reality-virtuality continuum, which clarifies the relationships among virtual reality (VR), augmented reality (AR), and mixed reality [10-12]. In this review, we use VR to denote predominantly computer-generated environments experienced with varying levels of immersion (eg, head-mounted displays or projection-based systems), AR to denote digital overlays and cues integrated into the real world (often via mobile or wearable devices), and CAVE (Cave Automatic Virtual Environment) systems as a projection-based, room-scale configuration of VR that supports embodied interaction and navigation without headgear [10,11,13]. Distinguishing these modalities is practically relevant because they differ in interaction demands, supervision requirements, space and cost constraints, and potential adverse effects, which can influence both feasibility and outcomes in older adults with cognitive impairment [14-16].

Across cognitive rehabilitation, immersive tasks have been used to target memory, attention, executive functions, and spatial or navigation abilities, and they can embed cognitively demanding activities within ADL or IADL-like scenarios to enhance ecological validity and functional relevance [13,17-19]. However, translation beyond controlled settings remains constrained by substantial heterogeneity in platforms and immersion levels, interaction modalities, task designs, intervention dose (session duration and frequency), comparator conditions, and outcome selection, which complicates synthesis and limits the strength and generalizability of inferences [4,20,21]. These challenges are amplified by the fact that XR-based outcomes cannot be interpreted independently of feasibility; usability, acceptability, and accessibility directly shape adherence, delivered exposure (effective “dose”), safety, and the interpretability of cognitive and functional measures in cognitively impaired older adults [14,15].

To address this, this review adopts an explicit usability lens. We use usability in the human-computer interaction sense as a multidimensional construct encompassing effectiveness, efficiency, and satisfaction in a specified context of use. In this literature, usability evidence may be drawn from standardized instruments, task performance indicators (eg, errors and assistance needs), completion and adherence metrics, qualitative user-experience reports, and stakeholder perspectives (patients, caregivers, and clinicians) [18,22]. This framing is particularly important in dementia and MCI because age- and disease-related sensory, motor, and cognitive constraints can make interface complexity, onboarding demands, fatigue, and tolerability decisive determinants of whether an intervention is deliverable, interpretable, and scalable [8,14,15].

Several systematic reviews have examined technology-supported cognitive rehabilitation, but the evidence base remains fragmented with respect to target population (MCI vs dementia or AD), XR modality (VR-only vs broader XR), and the depth and standardization of usability and implementation synthesis [3,4,20,23-27]. In parallel, broader assistive-technology reviews in dementia synthesize important adjacent evidence (including immersive and wearable technologies) but do not provide a modality-explicit, rehabilitation-focused synthesis that treats usability and translation constraints as analytically coded outcomes [28]. Likewise, AR-focused reviews for cognitively impaired populations emphasize action assistance and learning, but their scope is not centered on cognitive rehabilitation outcomes across the MCI-dementia continuum and does not integrate projection-based immersive configurations, such as CAVE [29]. Taken together, these gaps motivate a consolidated systematic review that (1) spans the MCI-dementia continuum, (2) compares immersive technology types with explicit attention to VR (including CAVE as projection-based VR) and AR, and (3) treats usability and implementation constraints as first-class evidence streams alongside cognitive and functional outcomes [14-16].

Accordingly, this review is guided by the following research question: What is the current evidence on usability, feasibility, and reported intervention outcomes of VR, AR, and CAVE systems for cognitive rehabilitation in individuals with MCI and dementia? In addressing this question, we additionally examine how intervention characteristics (technology type, immersion level, interaction demands, and dose), study design choices, and outcome selection contribute to heterogeneity in reported findings and shape the feasibility of translation beyond controlled settings [20,21,30].

## Methods

### Review Contributions and Structure

First, it synthesizes current evidence on the usability, therapeutic efficacy, and limitations of immersive systems (VR, AR, and CAVE) in cognitive rehabilitation for individuals with MCI and dementia.

Second, it compares approaches across technologies, study designs, and outcome measures to identify key trends and limitations in the field.

Finally, it outlines challenges and proposes priorities for future research, including methodological standardization and scalability of interventions.

**Figure 1.**
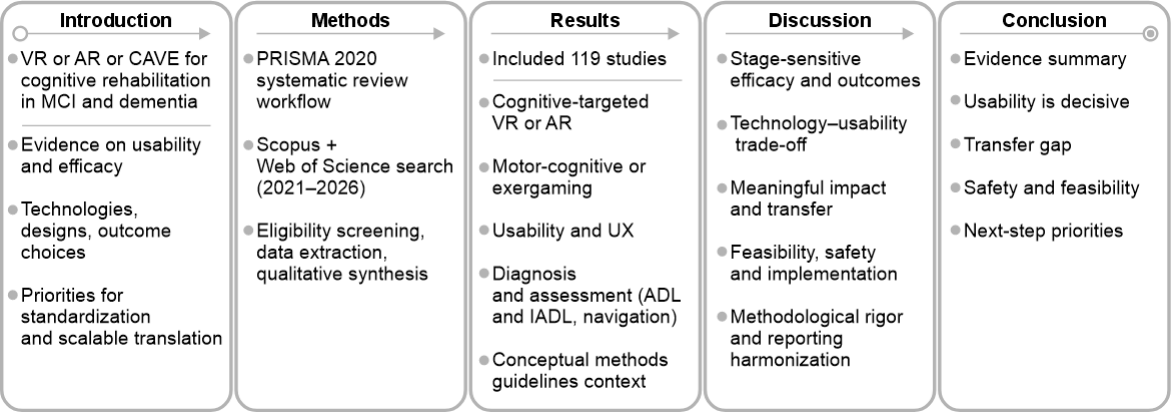
Graphical outline of the study. ADL: activities of daily living; AR: augmented reality; CAVE: Cave Automatic Virtual Environment; IADL: instrumental activities of daily living; MCI: mild cognitive impairment; PRISMA: Preferred Reporting Items for Systematic Reviews and Meta-Analyses; UX: user experience; VR: virtual reality.

The overall structure of this systematic review is summarized in Figure 1. After the Introduction section, the Methods section details the search strategy, eligibility criteria, study selection, data extraction, and synthesis approach. The Results section then reports the PRISMA (Preferred Reporting Items for Systematic Reviews and Meta-Analyses)-based selection process and presents the included evidence across VR, AR, and CAVE modalities. Next, the Discussion section interprets the findings with an emphasis on usability, feasibility, and translational considerations and highlights implications for research and practice. Finally, the Conclusions section summarizes the key takeaways and priorities for future work.

### Protocol and Registration

This systematic review was conducted in accordance with the PRISMA 2020 guidelines. The protocol was prospectively developed to ensure methodological rigor and transparency. Trial registration was not applicable, as this work did not involve primary clinical trials.

### Eligibility Criteria

Studies were considered eligible if they addressed the use of immersive technologies—specifically VR, AR, or CAVE systems—in the context of cognitive rehabilitation for individuals with MCI and/or dementia. The literature search was limited to publications from 2021 to 2026 and used the following query, “(virtual reality OR augmented reality OR CAVE) AND cognitive AND (dementia OR MCI) AND rehabilitation.” Only peer-reviewed journal articles written in English were included. Records without a DOI, missing author information or source title, or those that could not be accessed in full-text form were excluded. Conference papers, abstracts, editorials, and other nonarticle document types were removed. Furthermore, records deemed unrelated to cognitive rehabilitation, MCI, or dementia upon title and abstract screening were excluded. The final set of eligible studies was subsequently divided into 2 categories: that review articles and regular (original research) articles.

### Information Sources and Search Strategy

The literature search was conducted using 2 major scientific databases—Web of Science (WoS) and Scopus. These databases were selected due to their broad coverage of peer-reviewed journals in the fields of health care, rehabilitation, neuroscience, and immersive technologies. A structured search strategy was applied in both databases using a consistent combination of keywords and Boolean operators to identify relevant studies. The search focused on titles, abstracts, and author keywords and was designed to capture publications addressing the application of VR, AR, or CAVE systems in cognitive rehabilitation for individuals with MCI and/or dementia. The same search query and inclusion limits were applied across both databases to ensure comparability and reproducibility of the results.

### Selection Process

A total of 2 reviewers (RAS and JK) independently screened titles, abstracts, and full texts against predefined criteria. Disagreements were resolved through consensus or consultation with a third reviewer (MP). The selection process was documented using the PRISMA 2020 flow diagram.

### Data Extraction

Data were extracted using standardized forms capturing: study design, authorship, publication year, country, participant characteristics, intervention specifics (device type, duration, frequency, and context), cognitive domains targeted, and outcome measures (cognitive tests, usability scales, and emotional or behavioral outcomes). Extraction was performed in duplicate by independent reviewers to minimize errors.

### Risk of Bias and Quality Considerations

We performed a structured qualitative appraisal of methodological quality and potential sources of bias during data extraction and narrative synthesis. For each included publication, we recorded study design features and reporting characteristics that most strongly influence interpretability and transferability in immersive-technology research, including (1) study design and the presence or absence of comparator conditions, (2) sample size and participant characterization, (3) intervention or system description and dose (duration, frequency, and setting), (4) completeness and transparency of outcome reporting (including usability or acceptability metrics and adverse events such as cybersickness), (5) follow-up duration, and (6) practical implementation factors (equipment constraints, supervision requirements, staffing, and caregiver involvement). These quality considerations were used to contextualize findings, identify recurring limitations across studies, and guide cautious interpretation of reported outcomes in the narrative synthesis.

### Data Synthesis

Given heterogeneity in technologies, intervention content, and outcome measures, a meta-analysis was not feasible. We therefore conducted a narrative synthesis structured by technology type (VR, AR, and CAVE) and outcome domain (usability or acceptability, cognitive performance, daily functioning, and emotional well-being). Within each category, we summarized intervention and system characteristics (eg, hardware setup, immersion level, interaction modalities, delivery setting, and dose where reported) and synthesized reported outcomes qualitatively. For studies that included comparator conditions, we described the direction and consistency of reported between-group findings without statistical pooling. We also synthesized implementation-relevant factors, including onboarding demands, supervision requirements, and adverse events (eg, cybersickness) when reported.

## Results

### Literature Selection

This section was conducted in accordance with the PRISMA guidelines. Studies were included if they met clearly defined eligibility criteria, that are (1) publication period from 2021 to 2026, (2) English language, (3) peer-reviewed journal articles only, and (4) a clear focus on cognitive rehabilitation in individuals with MCI and/or dementia. Searches were conducted on November 2, 2025. Eligible interventions involved immersive or semi-immersive technologies, specifically VR, AR, or CAVE systems. Records without a DOI, missing author information or source title, or without accessible full-text availability were excluded, as were studies unrelated to cognitive rehabilitation, MCI, or dementia.

Comprehensive electronic searches were conducted in 2 major bibliographic databases, that is, Scopus and WoS. The search strategy used Boolean operators and the following search string, (virtual reality OR augmented reality OR CAVE) AND cognitive AND (dementia OR MCI) AND rehabilitation. The same search strategy and filters were applied consistently across both databases. Retrieved records were exported to reference management software, where duplicates were removed before screening.

Titles and abstracts were screened to exclude clearly irrelevant records, followed by full-text assessment of the remaining articles against the predefined inclusion and exclusion criteria. Only articles that could be downloaded in full text were retained. The final set of included studies was subsequently divided into 2 categories—review articles and regular (original research) articles, to enable structured comparison across study types.

Due to heterogeneity in study designs, intervention characteristics, and outcome measures, a meta-analysis was not feasible. Instead, a narrative synthesis was performed, focusing on the characteristics of the VR, AR, or CAVE interventions, targeted cognitive domains, reported outcomes related to rehabilitation and usability, and identified methodological limitations across studies.

**Figure 2.**
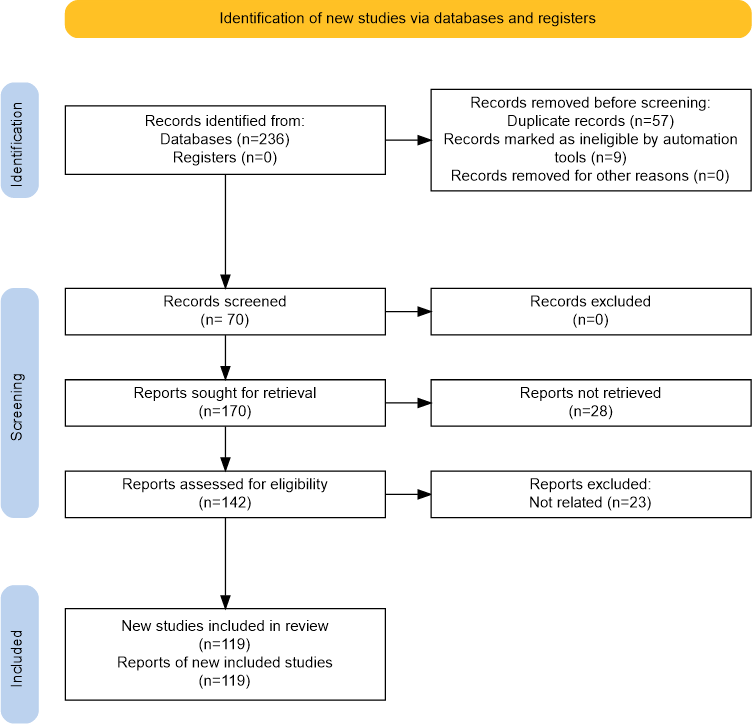
PRISMA (Preferred Reporting Items for Systematic Reviews and Meta-Analyses) 2020 flow diagram illustrating the article selection process.

The PRISMA flow diagram (Figure 2) summarizes the study selection process. A total of 236 records were identified from databases (Scopus and WoS), with no records from registers (n=0). Before screening, 57 duplicate records and 9 records marked as ineligible by automation tools were removed, leaving 170 records for screening [31]. No records were excluded at the screening stage (n=0); therefore, 170 reports were sought for retrieval, of which 28 reports could not be retrieved. The remaining 142 reports were assessed for eligibility, and 23 reports were excluded as not related. Ultimately, 119 studies [2-8,13-21,23-27,30-127] were included in the review (119 reports of included studies).

### Positioning of This Review Relative to Existing Systematic Reviews

A growing number of systematic reviews have already examined XR-supported cognitive rehabilitation, but their scopes remain fragmented with respect to target population, XR modality, immersion level, and the depth of usability and implementation synthesis. Several focus primarily on MCI and VR-based cognitive rehabilitation, often emphasizing cognitive efficacy and protocol characteristics while providing only limited, nonstandardized coverage of usability, feasibility, and real-world deployment constraints [3,4,20,23-27]. Other reviews address dementia, AD, or functional outcomes (ADL or IADL) and ecological validity, yet typically remain centered on VR (frequently non- or semi-immersive) and treat usability, acceptability, adverse events, and service integration as secondary considerations rather than as analytically coded dimensions [5,6,17]. In parallel, more specialized reviews concentrate on narrower constructs—such as technology-assisted dual-task rehabilitation or spatial navigation paradigms—providing valuable conceptual framing but not offering an implementation-oriented synthesis of immersive therapeutic XR across the MCI-dementia continuum [13,19]. Finally, several systematic reviews adopt broader clinical populations or technology umbrellas (eg, survivors of stroke, Parkinson disease, acquired cognitive disorders, or general digital-technology interventions in older adults), which supports methodological insight but limits direct transferability to dementia-focused cognitive rehabilitation pathways and to cross-modality comparisons including AR and CAVE [7,32-39,128]. A related strand reviews fully immersive VR-based cognitive remediation in adult psychiatric or psychosocial rehabilitation (including some MCI and mild dementia samples), highlighting intervention-component reporting gaps that constrain real-world implementation [40]. In addition, reviews outside the XR rehabilitation core (eg, bibliometric mapping in pediatrics or topic-distant clinical domains) further illustrate how rapidly expanding evidence can become fragmented and difficult to translate to dementia-specific implementation decisions [41-43].

Beyond cognitive-rehabilitation–centered syntheses, the XR literature also contains systematic reviews that cluster around 2 adjacent themes which inform, but do not substitute for, our focus on immersive XR (VR, AR, or CAVE) cognitive rehabilitation in MCI and dementia.

Reviews in the category “Psychosocial, Emotional, and Behavioral Interventions Using Immersive Technologies” predominantly frame immersive and digital technologies as instruments for psychosocial support, emotional well-being, or broader behavioral outcomes rather than as structured cognitive rehabilitation protocols targeted to the MCI-dementia continuum. For example, one line of work synthesizes XR use in older adults at a general level (including engagement and experience-oriented outcomes) without restricting scope to cognitive rehabilitation in MCI or dementia or to immersive XR modality comparisons [8]. Other reviews adopt an even broader “aging and well-being” perspective, discussing strategies to improve quality of life across normal versus pathological aging, which is informative for contextual motivation but not specific to immersive XR cognitive rehabilitation efficacy or implementation in dementia care pathways [44]. Several reviews examine psychosocially oriented digital interventions and symptom-focused cognitive supports (rather than immersive XR rehabilitation protocols), which limits transferability to our modality-specific and deployment-oriented synthesis in dementia or MCI [45]. A related strand focuses on reminiscence-oriented applications using VR-based approaches, which aligns with person-centered care and engagement, yet differs from our emphasis on cognitive rehabilitation outcomes and cross-technology synthesis across VR, AR, or CAVE [46]. Some reviews concentrate on specific training paradigms or outcome contexts—such as attention rehabilitation via serious games, or changes in cognitive screening performance across training regimes—where dementia-focused immersive XR rehabilitation and implementation or scalability considerations are not the primary analytic axis [47]. Importantly, additional reviews in this group address adjacent rehabilitation or stimulation concepts (eg, multisensory stimulation in dementia rehabilitation) that overlap conceptually but do not provide the XR-modality–differentiated usability and implementation synthesis that our review targets [48]. Finally, reviews focused on digital cognitive interventions in nondementia neurological populations (eg, traumatic brain injury) are methodologically relevant but population-mismatched for the MCI or dementia rehabilitation pathway that defines our eligibility boundaries [49]. Taken together, these help justify the broader psychosocial value and acceptability of immersive or digital interventions, but our review deliberately differentiates itself by restricting the population to MCI and dementia, expanding the technology scope to immersive XR (VR, AR, or CAVE), and systematically integrating usability and real-world implementation or scalability evidence alongside cognitive rehabilitation outcomes.

Reviews in the category “Design, Usability, Safety, and Implementation of Immersive Systems for Older Adults” are closer to our aims in that they foreground design, usability, safety, feasibility, and translational barriers; however, they commonly span heterogeneous populations, modalities, and use cases that dilute direct inference for dementia or MCI cognitive rehabilitation with immersive XR. For instance, a dedicated synthesis of cybersickness and fully immersive VR provides essential safety framing; yet, it is not specifically anchored in dementia or MCI cognitive rehabilitation workflows or in VR, AR, or CAVE cross-modality comparison [50]. Other work reviews XR technologies or interface classes relevant to implementation (eg, smart glasses for cognitive impairment), but it typically emphasizes device feasibility and adoption rather than mapping therapeutic cognitive rehabilitation effects across immersive XR modalities [51]. Several reviews focus on assessment- and function-oriented constructs (eg, IADL assessment) that are highly relevant to outcome selection, while still differing from our rehabilitation-centric scope and our modality-explicit synthesis across immersive XR systems [52]. Additional reviews address intervention families that are broader than immersive XR (eg, digital game-based or computer-based cognitive interventions in cognitively impaired community-dwelling populations), which informs feasibility and design considerations but does not resolve the immersion- and modality-specific trade-offs central to our review [53]. Similarly, comparative or umbrella-type syntheses of nonpharmacologic interventions in older adults provide important taxonomic and implementation context, yet they do not isolate immersive XR (VR, AR, or CAVE) cognitive rehabilitation for MCI or dementia as the analytic core [54]. Bibliometric and mapping-type reviews contribute useful evidence on research trends and gaps, but they do not offer the structured, outcome- and usability-centered comparative synthesis required to guide scalable deployment of immersive XR cognitive rehabilitation [55]. Finally, several reviews in this category focus on other clinical indications or technology ecosystems (eg, poststroke cognitive impairment, Parkinson disease, and sensor or interaction technologies), which provide transferable implementation lessons but remain population- or aim-mismatched to dementia or MCI cognitive rehabilitation [56]. Therefore, while these provide critical inputs for human-centered design, safety, and adoption, our review distinguishes itself by (1) focusing specifically on MCI and dementia, (2) treating immersive XR modality (VR, AR, and CAVE) and immersion level as explicit comparative dimensions, and (3) linking usability or feasibility and implementation or scalability evidence directly to cognitive rehabilitation outcomes and methodological standardization needs in real-world care settings.

### Therapeutic VR or AR Interventions Primarily Targeting Cognitive Functions

Table 1 summarizes therapeutic interventions in which the primary target is cognitive functioning, spanning neurodegenerative conditions (AD or dementia and Parkinson disease), MCI, poststroke cognitive impairment, and acquired brain injury, as well as prevention-oriented programs in older adults [57-65]. Across this body of work, interventions aim to improve global cognition and/or specific domains such as executive functions, memory (including working and prospective memory), attention, and visuospatial abilities through structured, task-based training embedded in virtual or augmented environments [66-70].

**Table 1.** Therapeutic VR^a^ or AR^b^ interventions primarily targeting cognitive functions.

Study (year)	Population or stage	Design and sample (N)	Technology and intervention	Dose and key cognitive outcomes
Lanzoni et al (2022) [69]	Adults with retrograde amnesia after brain stroke	Usability or design N=NR^c^	VR—Nonimmersive (screen) customizable serious games built from 3D scans of familiar environments for memory rehabilitation	Dose: Single or few sessions for usability testing Domains: Spatial memory, episodic memory, orientation Outcome: Demonstrated feasibility and usability; cognitive outcomes exploratory only
Oliveira et al (2021) [72]	Older adults with mild-to-moderate Alzheimer dementia	Pilot RCT^d^ N=17	VR—Immersive (HMD^e^ or CAVE^f^) VR cognitive stimulation reproducing IADL^g^	Dose: 2 months, 10 sessions (2 sessions/week) Domains: Global cognition, memory, attention, and executive functions Outcome: Improved global cognition with large effect size in the VR group compared with control
Ip et al (2025) [71]	Community-dwelling older adults with MCI^h^	Pilot RCT N=18	VR exergame—Nonimmersive (large screen) full-immersive VR game-based cognitive-motor training compared with group-based Baduanjin exercise	Dose: 8 weeks, 16 sessions (2 sessions/week) Domains: Executive functions, global cognition, and dual-task performance Outcome: Greater improvements in cognitive-motor performance, mobility, balance, and gait speed in the VR group; no clear benefit in executive function
Huang and Yang (2022) [58]	Older adults with dementia in long-term care	Observational N=20 (7 followed at 3-6 months)	VR—Immersive (HMD) VR reminiscence therapy using immersive scenes related to participants’ life history	Dose: 3 months, 2 sessions/week Domains: Global cognition (secondary), autobiographical memory, and mood Outcome: No significant cognitive changes immediately post intervention, but depressive symptoms decreased; cognition declined again at 3-6 months
De Simone et al (2023) [60]	Patients with Parkinson disease and MCI; healthy older adults	Protocol N=60 (30 PD-MCI and 30 healthy)	VR+telemedicine—Immersive (HMD) home-based VR cognitive training targeting executive functions to improve prospective memory	Dose: Planned multiweek intervention (pre- and post–2-month follow-up) Domains: Prospective memory and executive functions (planning, shifting, and updating) Outcome: Only baseline and feasibility results available; cognitive efficacy not yet reported
Han et al (2021) [67]	Older adults at risk of cognitive decline (community dwelling)	Prototype and expert evaluation; exploratory effectiveness N=NR	AR—Mobile AR gamified AR cognitive training system inspired by Trail Making Test, performed in real space	Dose: Short-term pilot (single or few sessions) Domains: Working memory, attention, and visual perception Outcome: Experts judged that the system could improve working memory and attention; effectiveness in older adults not yet fully tested
De Luca et al (2024) [62]	Adults with severe acquired brain injury (traumatic and nontraumatic)	RCT N=40 (20 VR-based ROT^i^, 20 control)	VR—Nonimmersive (screen) VR-based ROT compared with standard reality orientation therapy	Dose: Inpatient rehabilitation cycle (several weeks) Domains: Global cognition (MMSE^j^), orientation, and attention Outcome: Both groups improved, but VR-ROT showed greater MMSE gains and reduced depressive symptoms
Buele et al (2024) [66]	Older adults with MCI	Single-blind RCT N=34 (17 VR, 17 control)	VR—Semi or full immersive group motor training followed by VR-based cognitive training vs motor training plus traditional cognitive training	Dose: 6 weeks, 12 sessions (40 minutes each) Domains: Global cognition, executive functions, and memory Outcome: Both groups improved in cognition and depression; between-group differences were not significant, but VR group reached higher task difficulty
Downey et al (2023) [64]	Middle-aged and older adults with and without hearing loss	Single-blind RCT N=NR	CCT^k^+VR assessment—Immersive (assessment) At-home computerized executive-function training vs wait-list control, with dual-task gait	Dose: 12 weeks of at-home training Domains: Executive functions, attention, and dual-task performance Outcome: Planned to test whether executive function training improves cognitive-motor dual-task performance; primary outcomes not fully reported yet
Liu et al (2023) [61]	Older adults with poststroke MCI	Pilot RCT N=30 (15 VR, 15 control)	VR—Immersive (HMD) immersive VR puzzle games added to routine rehabilitation vs traditional cognitive training	Dose: 6 weeks of treatment Domains: Executive function, visuospatial attention, and processing speed Outcome: Significant improvement in DSST in VR group, suggesting gains in executive and visuospatial functions; other measures improved but not sign…
Oliveira et al (2021) [72]	Institutionalized nonrobust older adults	Single-blind RCT N=NR	VR exergame—Nonimmersive (exergame) 2D VR exercise training vs equivalent exercises without VR	Dose: 2 months, 16 sessions (2 sessions/week) Domains: Spatial navigation and executive function Outcome: VR group showed significantly greater improvement in spatial navigation compared with active control
Jeong et al (2023) [68]	Adults with acquired brain injury	Randomized crossover trial N=NR	VR (music)—Immersive (HMD) VR-based music attention training (drumming tasks) vs conventional computerized cognitive training	Dose: Two 4-week phases (VR vs control) in crossover Domains: Selective, alternating, and divided attention; executive function Outcome: VR-MAT improved attention and some executive measures more than conventional training in at least 1 phase
Maeng et al (2021) [70]	Older adults with and without MCI	RCT N=NR	VR—Nonimmersive VR-based cognitive training tasks (eg, ADL^l^ scenarios and puzzles) compared with conventional or no training	Dose: Multiweek program with repeated sessions Domains: Global cognition, memory, executive functions, and attention Outcome: Improvement or stabilization of cognition in older adult participants, particularly those with MCI
Baldimtsi et al (2023) [63]	Older adults with MCI	Randomized intervention trial N=NR	VR (physical+cognitive)—Nonimmersive (likely) combined physical exercise and VR-based cognitive tasks vs conventional or usual care	Dose: Several weeks with multiple sessions per week Domains: Executive functions, memory, and attention Outcome: VR physical+cognitive training associated with greater cognitive gains than comparison conditions
Fiorenzato et al (2025) [59]	Patients with Parkinson disease and healthy older adults	RCT N=NR	VR—Nonimmersive VR-based cognitive training tasks targeting prospective memory and executive functions	Dose: Multisession intervention over several weeks Domains: Executive functions and prospective memory Outcome: Improvements in prospective memory and executive functions in both PD and healthy aging groups
Zhu et al (2022) [65]	Adult patients undergoing laparoscopic surgery	Protocol N=NR	VR—Immersive (HMD) biophilic VR exposure pre- and/or postoperatively compared with sham VR	Dose: Short perioperative VR sessions Domains: Postoperative cognitive function (attention, working memory, and global cognition) Outcome: No results yet; hypothesis that biophilic VR will mitigate postoperative cognitive decline
Początek et al (2023) [73]	Patients with Alzheimer disease	Narrative or systematic review N=NR	Mixed (including VR or AR)—not applicable, overview of cognitive training approaches, including emerging VR-based methods	Dose: Various across included trials Domains: Memory, executive functions, language, and attention Outcome: Cognitive training yields small-to-moderate benefits; VR or AR highlighted as promising but underresearched
Cavallo and Lasaponara (2025) [74]	Patients with various neurodegenerative diseases (eg, Alzheimer disease and Parkinson disease)	Narrative review N=NR	Mixed (including VR or AR)—not applicable, overview of cognitive deficits and cognitive training modalities in neurodegenerative diseases	Dose: Various across discussed studies Domains: Multiple domains (memory, executive functions, attention, and visuospatial abilities) Outcome: Supports use of structured cognitive training; notes need for more high-quality RCTs and the potential of VR or AR

a VR: virtual reality.

b AR: augmented reality.

c NR: not reported.

d RCT: randomized controlled trial.

e HMD: head-mounted display.

f CAVE: Cave Automatic Virtual Environment.

g IADL: instrumental activities of daily living.

h MCI: mild cognitive impairment.

i ROT: reality orientation therapy.

j MMSE: Mini-Mental State Examination.

k CCT: computerized cognitive training.

l ADL: activities of daily living.

Most studies prioritize executive functioning and attentional control as core therapeutic targets, reflecting their relevance for everyday functioning and their frequent impairment in clinical aging and neurological populations [59,60,62,68]. Memory-focused approaches include working memory training (notably via mobile AR) and prospective memory training in Parkinson disease and healthy aging, with several programs attempting to increase ecological validity by using realistic or meaningful task contexts [57,59,67].

In poststroke and acquired brain injury contexts, VR is used both for domain-specific cognitive rehabilitation (eg, visuospatial attention and executive processes) and for broader orientation and behavioral support, often as an adjunct to standard rehabilitation [61,62].

The subgroup includes immersive head-mounted display (HMD)–based VR (eg, reminiscence and cognitive training), nonimmersive or screen-based VR platforms, and mobile AR serious games [58,61,67,69,70]. A notable direction is the integration of immersive VR with telemedicine or home-based delivery concepts intended to improve accessibility and scalability for older adults and patients with mobility constraints [60]. In contrast, several studies remain clinic- or laboratory-based and emphasize feasibility or usability, platform design, and therapist-friendly monitoring rather than definitive efficacy [67,69,70]. Home-oriented delivery is also reflected in at-home computerized executive-function training designs that use immersive VR environments as ecologically valid contexts for dual-task assessment of cognition and mobility, extending evaluation beyond standard clinic-based testing [64].

Methodological rigor varies substantially, ranging from pilot and single-blind randomized controlled trials to crossover designs and observational follow-up studies [57,58,66,68]. Control conditions include traditional cognitive training, standard rehabilitation or orientation therapy, and non-VR exercise programs, which help isolate VR or AR-specific contributions but also introduce heterogeneity in active ingredients and “dose” [61,62,66,71]. Outcome batteries typically combine global cognitive screening tools with domain-specific neuropsychological tests (executive function, attention, and memory), and—less consistently—functional end points (ADL or IADL), mood, or user experience (UX) measures [57,58,62,66]. Of the total, 2 included articles provide a broader conceptual and methodological context for cognitive training in neurodegenerative disease, highlighting persistent challenges around outcome heterogeneity, transfer to daily functioning, and evidence quality [73,74].

Across populations, many studies report favorable feasibility indicators (acceptability, adherence, and tolerability) and within-group improvements on at least some cognitive measures, particularly when interventions are engaging, adaptive, and anchored in meaningful tasks [61,66,68,70]. However, between-group superiority is not uniform, and several studies are explicitly positioned as pilots or preliminary reports, limiting statistical power and confidence in effect estimates [57,58,60,61]. Reminiscence-oriented immersive VR appears especially relevant for affective and engagement-related outcomes in dementia, with cognitive effects that may be modest and time-limited without maintained intervention [58]. For MCI, combined or multicomponent programs (eg, motor plus VR-based cognitive training) reflect a practical clinical strategy, although gains in daily functioning are not consistently observed [66,71].

Overall, the subgroup supports the feasibility of delivering cognitively oriented interventions using VR or AR across diverse older and neurological populations, but comparability is constrained by heterogeneity in immersion level, task design, intervention dose, and outcome selection [57,59,67,70]. To strengthen future quantitative synthesis, priorities include (1) harmonized core outcome sets (global cognition+domain-specific measures+functional end points), (2) standardized reporting of intervention dose and fidelity, (3) longer follow-up to assess durability, and (4) clearer mapping between task mechanics and hypothesized transfer targets [73,74]. Finally, protocol-driven work targeting cognitive vulnerability in perioperative settings illustrates ongoing diversification of VR applications, but such studies should be clearly separated from dementia or MCI rehabilitation pathways when interpreting population-specific clinical relevance and implementation needs [65].

### Motor-Cognitive and/or Exergaming VR Interventions in Neurorehabilitation

Table 2 summarizes studies in which VR-based approaches combine explicit cognitive demands with concurrent motor engagement (eg, stepping, balance training, cycling, or exergaming) and are applied in neurorehabilitation-relevant contexts. The included evidence spans community-dwelling older adults with cognitive frailty [75], individuals with MCI [76,77], mild AD (protocol) [78], and poststroke cognitive disorders [79,80]. Overall, delivery most often relies on nonimmersive or semi-immersive setups, typically screen-based interaction combined with motion tracking or instrumented exercise equipment [81].

**Table 2.** Motor-cognitive and/or exergaming VR^a^ interventions in neurorehabilitation.

Study (Year)	Population or stage	Design and sample (N)	Technology and intervention	Dose and key cognitive outcomes
Kwan et al (2024) [75]	Community-dwelling older adults with cognitive frailty (physical frailty+MCI^b^), no dementia	Multicenter, assessor-blinded parallel-group RCT^c^ (VRMCT^d^ vs usual care); N=293 (≈146 VRMCT, 147 control)	Nonimmersive VR motor-cognitive exergame platform with motion sensing; nonimmersive group-based VR motor-cognitive exercises; 16 one-hour sessions led by interventionists	16×60 min; 2 times per week; 8 weeks Domains: Global cognition, executive function, memory, interference control VRMCT significantly improved global cognitive function vs usual care; some benefits in executive measures
Fu and Wang (2025) [76]	Patients with MCI treated in a neurology department	Randomized controlled clinical observation (VR+acupuncture vs VR only); N=48 (24 VR+acupuncture; 24 VR only)	VR rehabilitation training system with virtual scenes plus acupuncture in treatment arm; Non-immersive; Control: VR training only; Treatment: same VR program plus scalp/eye acupuncture during each session	VR training protocol described qualitatively; acupuncture needles retained ~30 min each session Domains: Global cognition (MMSE^e^, MoCA^f^), daily living skills Treatment group showed larger gains in MMSE and MoCA and higher overall efficacy vs VR-only control
De Luca et al (2022) [82]	Single patient with Nasu–Hakola disease (TREM2-related dementia) and motor-cognitive deficits	Single-case crossover (conventional rehabilitation phase vs conventional + VRRS^g^ phase); N=1	VRRS with cognitive and physical modules; Nonimmersive Phase 1: standard physical + cognitive training; Phase 2: 30 speech + 30 PT sessions plus 60 VRRS sessions (cognitive + motor)	VRRS phase: 3 sessions/week, 60 min; total 60 VRRS sessions after 12 weeks of conventional rehab. Domains: Attention, memory, executive function; affective symptoms Improved cognitive tests and marked reduction in anxiety, apathy, indifference, and depressive symptoms after VRRS phase
Huber et al (2024) [80]	Adults in chronic phase after stroke with residual motor or cognitive deficits	Single-blind parallel-group RCT protocol (exergame + usual care vs usual care); Target N=38 chronic survivors of stroke	Concept-guided personalized motor-cognitive exergame training using video game–based tasks; Nonimmersive Intervention group performs exergame training in addition to usual care; control continues usual care only	2 times per week; 30-40 min; 12 weeks (24 sessions) Domains: Global cognition (MoCA) and domain-specific cognitive functions Results pending; expected superior improvement in cognition and gait in exergame group
Faria et al (2023) [83]	Adults with acquired brain injury, dementia, or age-related cognitive decline (conceptual target)	Methods or architecture paper (no clinical trial); Not applicable	VR-based activities-of-daily-living simulations integrated with AI-driven user profiling and adaptation; Non- or semi-immersive VR ADL environments Framework where VR ADL tasks are adapted in real time using AI models to personalize neurorehabilitation	Not fixed; example scenarios only (conceptual) Domains: Executive function, attention, memory, functional cognition No empirical outcomes; framework is intended to support individualized improvement in functional cognition
Manenti et al (2024) [77]	Adults with MCI from multiple Italian centers	Multicenter randomized active-controlled trial with five treatment arms; N=109 participants with MCI allocated to 5 groups	Clinic- and home-based VRRS cognitive training combined with anodal or sham tDCS^h^, or TAU; Non-immersive VR / telerehabilitation All active arms: 12 × 60-min face-to-face VRRS sessions; some arms also receive anodal tDCS and/or 12 weeks of home VRRS telerehabilitation or unstructured activity	Clinic VRRS: 12 × 60 min over 4 weeks; plus arm-specific telerehabilitation or unstructured… Domains: Episodic memory (primary), plus global cognition and other… VRRS + anodal tDCS followed by VRRS telerehabilitation produced the largest and most durable gains in episodic memory; VRRS-based protocols outperformed TAU
Maggio et al (2025) [84]	Informal caregivers of people with MCI	Nonrandomized single-arm pilot pre-post study; N=10 caregivers	Semi-immersive VR-based physical training program (K-HERO); Semi-immersive Individual low-intensity VR exercise sessions performed while accompanying relatives to rehabilitation	6 sessions of 30–40 min each during caregivers’ clinic visits Domains: No direct cognitive training; outcomes focus on psychological well-… Not applicable; reported reductions in avoidance and social-support–based coping and good usability
Hwang et al (2021) [81]	Community-dwelling older adults with age-related cognitive and gait concerns	Randomized controlled trial (VRCT+locomotor vs tabletop cognitive training); N=18 (9 VRCT^i^+locomotor, 9 control)	Semi-immersive VR cognitive training system combined with locomotor tasks; Semi-immersive experimental group performs VR cognitive tasks while stepping or locomoting; control receives tabletop cognitive activities with the same schedule	30 min/day, 3 times per week for 6 weeks (18 sessions) Domains: Processing speed, attention, working memory, and executive function VR group showed greater improvements in executive and attention measures (eg, TMT-A, Digit Span backward) vs control
Contrada et al (2025) [79]	Adults in the chronic phase after ischemic stroke with cognitive impairment	Within-subject longitudinal study (single group; pre, post, 6-month follow-up); N=18	Home-based multidomain cognitive training via VRRS Tele-NeuroRehabilitation platform; Nonimmersive VR or telerehabilitation Patients perform therapist-prescribed VRRS cognitive exercises at home with remote monitoring and feedback	5 sessions/week, 60 min each, for 4 weeks (20 sessions) Domains: Logical reasoning, attention, executive function, memory, language Significant improvements in working memory and language abilities and reduction in depressive symptoms, maintained at 6 months
Bourrelier et al (2021) [85]	Frail older adults with cognitive impairment (MMSE 15–28) and slow gait (<0.65 m/s)	Randomized controlled trial (VR balance training vs Nintendo Wii); N=22 analyzed (from 37 randomized)	VR balance-training system using game-like upper-limb and postural tasks; Likely semi-immersive (screen-based) Ten VR balance-training sessions vs ten Wii-bowling sessions over 3 weeks; APAs assessed using EMG	10 sessions in 3 weeks (~3-4 sessions/week); session length not clearly reported Domains: Not primarily cognitive; cognitive engagement through interactive… Main effects on postural adjustments; VR training improved APA timing and functional reach more than Wii
Gambella et al (2022) [78]	Community-dwelling adults with mild Alzheimer disease living at home with caregiver	Randomized controlled trial protocol (VR cycling + CCT^j^ vs standard bike + CCT); Planned N=78 (39 VR+CCT; 39 control)	jDome VR cycling system combined with computerized cognitive training; Semi-immersive VR (projected dome environment) Both groups receive computerized cognitive training; experimental group cycles in jDome VR, control uses standard exercise bike	16 × 60-min sessions; 2×/week over 8 weeks with 3-month follow-up Domains: Global cognition (MMSE, ADAS-Cog) plus quality of life, mood, behavior Outcomes not yet reported; aim is stabilization or improvement of global cognition in mild AD
Tang et al (2025) [86]	Adults from the general public with no dementia or caregiving history (empathy training target)	Controlled pre-post study (VR dementia tour vs control); N=80 adults (visual display terminal vs control)	Fully immersive VR dementia tour using interactive narrative persona; Fully immersive Single VR session simulating sensory and cognitive impairments of dementia; compared with non-VR control condition	Single VR session (duration not clearly specified) Domains: Not rehabilitation of patients; focuses on dementia knowledge and… VDT participants showed greater improvements in dementia knowledge and empathy compared with control

a VR: virtual reality.

b MCI: mild cognitive impairment.

c RCT: randomized controlled trial.

d VRMCT: virtual reality motor-cognitive training.

e MMSE: Mini-Mental State Examination.

f MoCA: Montreal Cognitive Assessment.

g VRRS: Virtual Reality Rehabilitation System.

h tDCS: transcranial direct current stimulation.

i VRCT: virtual reality cognitive training.

j CCT: computerized cognitive training.

Across intervention studies, the motor-cognitive rationale is commonly framed as training attention, working memory, and executive control under dual-task or enriched sensorimotor conditions. In a large multicenter randomized controlled trial among older adults with cognitive frailty, structured VR motor-cognitive training yielded improvements in global cognition compared with usual care [75]. Smaller trials in community-dwelling older adults further suggest that coupling cognitive tasks with locomotor activity can produce concurrent gains in cognitive performance and mobility-related measures (eg, walking speed), supporting the premise that integrated training may facilitate transfer to functional capacities [81]. Balance-oriented VR interventions target motor control processes that are sensitive to cognitive status; for example, VR-based training improved anticipatory postural adjustment timing relative to an active exergaming comparator (Nintendo Wii), indicating potential benefits for neuromotor preparation in frail older adults with cognitive and motor deficits [85].

A second theme concerns scalability, personalization, and continuity of care. Home-based telerehabilitation delivered via VR rehabilitation platforms with remote monitoring enables high-frequency practice and may support maintenance of gains in chronic poststroke populations [79]. Several contributions in this group are protocols rather than completed efficacy trials, reflecting ongoing consolidation of feasibility and methodological standards. The Physical Exercise and Mobile Cognitive Stimulation protocol, for instance, formalizes a concept-guided personalized motor-cognitive exergame program for chronic stroke, with global cognition (Montreal Cognitive Assessment [MoCA]) as a primary end point and gait or dual-task metrics as complementary outcomes [80]. In AD, the jDome protocol integrates VR-supported cycling with concurrent cognitive training to test whether adding a VR exercise context enhances outcomes beyond conventional exercise plus cognitive training [78]. Complementing these clinical studies, an AI-driven methodology paper proposes adaptive profiling and real-time task adjustment within VR-based ADLs scenarios to address heterogeneity in impairment severity and learning rates [83].

Some studies combine VR training with adjunct modalities, which may be clinically pragmatic but complicate causal attribution. In MCI, a multicenter randomized active-controlled study reported long-lasting improvements in episodic memory when VR-based cognitive treatment was combined with transcranial direct current stimulation and followed by telerehabilitation, suggesting that technology-enabled continuation at home may contribute to long-term effects [77]. Another clinical observation compared VR training alone with VR combined with acupuncture and reported larger improvements on screening measures (Mini-Mental State Examination [MMSE] or MoCA) in the combined arm [76]. In a rare neurodegenerative disease, a case report described a multimodal program incorporating VR rehabilitation and reported improvements across cognitive and neuropsychiatric measures, although the single-case design and concurrent therapies limit generalizability [82].

Several included publications provide implementation-relevant context rather than direct efficacy evidence. A cross-sectional analysis highlights the co-occurrence of cognitive impairment and psychological morbidity among survivors of stroke in rehabilitation, underscoring the need for scalable cognitive-motor rehabilitation approaches [87]. An opinion article summarizes recent VR developments for poststroke cognitive impairment and discusses practical considerations and limitations of the current evidence [88]. A national clinician survey similarly characterizes adoption, perceived value, and barriers to technology-enabled rehabilitation (including VR and telerehabilitation), informing feasibility and scalability considerations [89]. Beyond patient rehabilitation, an immersive “dementia tour” design study illustrates broader VR content development around dementia and highlights usability and engagement considerations that may be transferable to therapeutic design [86]. In the wider care ecosystem, a pilot study delivering low-intensity VR exercise to caregivers emphasized acceptability and potential psychosocial benefits, although it did not directly address patient cognitive outcomes [84].

In summary, motor-cognitive and exergaming VR interventions show promising signals across cognition- and mobility-relevant outcomes, but the evidence remains heterogeneous in platforms, immersion, intervention dose, and outcome selection [81,85]. The strongest inferences arise from larger randomized designs [75,77], whereas smaller trials, single-group telerehabilitation studies, and protocols primarily support feasibility and hypothesis generation [78-80]. Future work would benefit from harmonized cognitive end points beyond screening tools, explicit reporting of motor-cognitive task composition and progression, and longer follow-up to test durability and transfer to real-world functioning.

### Usability, Acceptability, and UX of VR or AR Systems

Across the included studies, usability and acceptability (Table 3) were treated as primary feasibility outcomes rather than secondary implementation considerations, reflecting the field’s current emphasis on early-stage validation of immersive and semi-immersive interventions. Evidence spans community-dwelling older adults and mixed-ability samples [90-92], clinical groups with MCI [18,93-98], AD [99], Parkinson disease [100], and dementia care contexts incorporating carers and professionals [101,102]. Collectively, these studies show that VR-based approaches are typically perceived as engaging and feasible when sessions are structured, onboarding is provided, and interaction demands are calibrated to users’ capabilities [18,90,91,93,94].

**Table 3.** Usability, acceptability, and UX^h^ of VR^b^ or AR^c^ systems.

Study (year)	Population or stage	Design and sample (N)	Technology and intervention	Dose or key cognitive outcomes
Chau et al (2021) [90]	Community-dwelling older adults and people with various disabilities; mixed physical and cognitive abilities	Single-arm pretest–posttest feasibility study; N=NR^d^	Non-immersive motion-tracking VR games targeting upper or lower limb movements, cognitive tasks, community-living skills, and relaxing scenes (exergaming)	Approximately 30-min sessions, 3 times per week for 6 weeks in community or service centers Physical and cognitive function, mood, quality of life, usage statistics, adverse events Preliminary improvements in some physical or cognitive outcomes; high acceptability and good adherence; few adverse events → VR training feasible for heterogeneous older and disabled
Chuang et al (2025) [103]	Community-dwelling older adults, mainly without diagnosed dementia but with age-related cognitive concerns	Single-arm pretest–posttest pilot study; N=NR	Fully immersive HMD^e^-based VR leisure and cognitive games; standing and seated interaction with light exergaming elements	Multiple VR sessions over several weeks in laboratory or clinic setting Cognitive performance (attention, memory, and executive function), self-reported enjoyment and usability, feasible and safe; participants enjoyed the intervention; some mild cybersickness or discomfort; preliminary cognitive improvements but no definitive efficacy conclusions
Kim et al (2025) [91]	Community-dwelling older adults, mostly cognitively intact or with mild decline	Single-arm feasibility and acceptability study; N=NR	Immersive HMD VR puzzle tasks requiring visuospatial problem-solving and manual interaction; low-to-moderate motor demands	One or several short VR sessions in a controlled environment Usability ratings, enjoyment, motion sickness, basic cognitive/engagement measures VR puzzles acceptable and engaging; older adults could use the system with guidance; main issues were headset comfort and controller complexity
Hassandra et al (2021) [93]	Older adults with MCI^f^	Mixed methods feasibility study (quantitative plus qualitative); N=NR	Semi-immersive VR exergame combining cycling or stepping with cognitive tasks (dual-task motor-cognitive training)	Repeated training sessions over several weeks in outpatient or rehabilitation setting Physical performance, cognitive outcomes, usability metrics, qualitative UX motor-cognitive exergaming feasible and acceptable for MCI; participants reported enjoyment and perceived benefit; some technical or usability issues;
Kua et al (2025) [94]	Older adult participants with MCI	Feasibility randomized pilot trial (VR vs control or alternative training); N=NR	Non- or semi-immersive VR tasks simulating everyday activities with cognitive focus and limited motor interaction	Multisession program over several weeks in a clinical setting; neuropsychological tests (memory, attention, and executive function) and feasibility indicators VR group showed at least comparable and, in some domains, better cognitive outcomes than control; acceptable adherence and dropout; mild fatigue or discomfort in some participants
Perra et al (2024) [96]	Older adults with MCI	Feasibility randomized controlled trial – study protocol; N=NR	VR-based cognitive remediation platform with everyday-like tasks and motor interaction (navigation and object manipulation)	Planned multiweek VR program with repeated sessions in clinical or outpatient setting Planned: cognitive function, daily functioning, quality of life, usability, adherence Protocol outlines rationale, design, and feasibility end points; emphasizes user-centered design, safety monitoring, and adherence for older adults with MCI
Latella et al (2024) [18]	Patients diagnosed with MCI	Single-arm feasibility and usability study; N=NR	Nonimmersive VR platform simulating everyday environments (eg, shopping and navigation) to train ecological cognitive skills; limited motor components	Series of VR sessions over several weeks in clinic or rehabilitation center Ecological cognitive performance, standardized cognitive tests, usability, and satisfaction Most participants with MCI could use VESPA^g^ 2; tasks perceived as ecologically valid; some technical difficulties and need for therapist guidance; early signals of improvement in ecolog…
Maggio et al (2025) [100]	Patients with mild-to-moderate Parkinson disease, with cognitive involvement	Pilot randomized controlled trial (VR telerehabilitation vs control or standard care); N=NR	Home-based VR telerehabilitation platform for cognitive tasks with simple motor interaction	Scheduled remote VR sessions at home over several weeks Cognitive outcomes, Parkinson-specific measures, quality of life, feasibility, and caregiver-related measures Telerehabilitation via VR was feasible and well-accepted; adherence adequate; cognitive outcomes showed promising trends; remote delivery reduced travel burden
Jeong et al (2025) [99]	Patients with mild-to-moderate Alzheimer disease	Single-arm preliminary feasibility study; N=NR	Nonimmersive VR cognitive training tasks (eg, navigation and memory) with simple motor interaction	Short multisession VR program in clinical or day-care setting Cognitive test scores, behavioral responses, usability indicators, adverse events VR training was feasible for selected patients with Alzheimer disease with adequate support; some had difficulties with navigation and task understanding; no serious adverse events;
Mondellini et al (2022) [95]	Older adults with MCI from Estonia and Italy	Cross-sectional comparative study between countries; N=NR	Immersive HMD VR cognitive task with mild motor demands (head or hand movements)	Single VR session in laboratory or clinic setting UX, presence, comfort, motion sickness, basic task performance Both groups completed the task; cultural and individual differences in UX and motion sickness; overall feasibility acceptable with supervision
Munoz et al (2025) [92]	Older adults with varying cognitive status using VR games	Observational data-driven study using classifier models; N=NR	VR exergame or cognitive game platform with motion-tracked head movements; focus on kinematic data rather than training efficacy	Single or repeated gaming sessions in controlled environment Head kinematics, subjective game UX, cognitive test scores Classifiers could link kinematic patterns to cognitive status and UX; supports feasibility of VR sensor data as digital biomarkers;
Stasolla and Di Gioia (2023) [97]	Individuals with mild neurocognitive impairment and their caregivers	Studying Usability and acceptability (conceptual and small sample); N=NR	Early-stage VR system where reinforcement learning adapts cognitive training parameters; some motor interaction	Limited number of sessions or system walkthroughs in clinic or laboratory Usability ratings, satisfaction, perceived usefulness, caregiver feedback Patients and caregivers generally accepted the RL-VR concept; main issues were technical or interface-related
Matsangidou et al (2023) [102]	People living with dementia in care settings	Participatory design study with pilot evaluation; N=NR	VR system for physical rehabilitation (movement tasks or games) with cognitive engagement; non- or semi-immersive	Iterative co-design workshops and pilot VR sessions in care facilities Qualitative feedback from patients, staff, caregivers; observations of engagement and physical activity Co-designed VR rehab matched abilities and preferences; participants could engage in guided VR tasks; staff noted motivational benefits and practical challenges
Gokani et al (2025) [101]	Informal carers and health or social care professionals working with people with dementia	Qualitative study using interviews or focus groups; N=NR	Various VR scenarios (often immersive or 360°) used for stimulation, reminiscence, and engagement	Discussion plus limited VR demonstrations: no fixed dose Perceived usefulness, risks, ethical issues, feasibility in routine care VR seen as promising for engagement and reminiscence; concerns about cost, motion sickness, staff training, and integration into routine care;
Chen et al (2025) [104]	Community-dwelling and/or institutionalized older adults, mixed cognitive status	Qualitative or mixed methods user-experience study; N=NR	Immersive VR experiences (sightseeing, nature, and simple interactive content) with low physical intensity	One or a few VR sessions in laboratory, community, or residential settings UX, presence, enjoyment, discomfort, perceived value Most older adults were curious and engaged; VR is generally acceptable, though some anxiety, disorientation, or cybersickness; stresses gradual introduction and ergonomic adjustments
Siette et al (2024) [105]	Adults and/or older adults using the LEAF CAF VR module	Think-aloud qualitative usability study; N=NR	VR training or assessment module with cognitive and functional tasks; interaction via controllers or gestures	Single-session interaction with the module in a laboratory Usability problems, interaction patterns, perceived workload, user feedback Identified specific usability barriers (interface complexity, confusing feedback, and navigation issues) and facilitators
Pedroli et al (2022) [106]	Adults or older adults with cognitive impairment using home-based training	Usability and acceptability pilot study; N=NR	Nonimmersive mobile or tablet application with structured cognitive exercises, possibly using enriched media but no full VR HMD	Home-based use over several weeks; self-administered with minimal or remote supervision Task completion, app usage, usability questionnaires, satisfaction Home-based app generally usable; adherence depended on support and motivation; participants appreciated flexibility; technical literacy and device issues were barriers
Tseng and Giau (2022) [98]	Older adults evaluating potential living environments	Feasibility and usability study; N=NR	Non- or semi-immersive VR walkthroughs of housing or environmental designs; focus on navigation and environmental fit	One or a few VR walkthrough sessions in laboratory or facility Perceived safety and usability, environmental preferences, motion sickness, orientation VR pre-occupancy evaluation was feasible; older adults could meaningfully comment on design features; some required support with navigation and controls
Ghosh et al (2022) [107]	Older adults with MCI participating in paired or social activities	Iterative user-centered design study with small pilots; N=NR	Robot-mediated interaction platform (non-VR) supporting cognitive and social activities with light motor components	Multiple design–test cycles with sessions in laboratory or care setting Usability, acceptability, engagement, perceived social and cognitive benefits Older adults with MCI could engage with robot-mediated paired tasks; emphasizes gradual complexity, clear feedback, and social aspects
Haley et al (2021) [108]	Adults with acquired apraxia of speech	Feasibility and therapeutic-effect pre-post study (sometimes with small comparison group); N=NR	Non-VR tablet or computer-based speech therapy program with autonomy-supportive features; motor speech rehabilitation	Multiple therapy sessions over several weeks in clinical setting Speech production measures, communication outcomes, adherence, and user satisfaction Feasible with positive therapeutic effects on speech measures; autonomy-supportive design enhanced engagement and motivation

a UX: user experience.

b VR: virtual reality.

c AR: augmented reality.

d NR: not reported.

e HMD: head-mounted display.

f MCI: mild cognitive impairment.

g VESPA: Virtual Environment for Spatial Processing Assessment.

Quantitative usability or acceptance assessments commonly relied on standardized questionnaires (most frequently the System Usability Scale and related acceptance models), with generally favorable perceptions reported by older end users in both community and clinical settings [91,93,94,103]. Importantly, perspectives sometimes diverged between stakeholders. For example, older adults tended to report high enjoyment and ease of use in fully immersive, leisure-based cognitive training, while professional raters judged overall usability more conservatively [103]. In technology-driven cognitive rehabilitation platforms, feasibility was often demonstrated through completion or adherence alongside positive usability ratings, supporting the practicality of repeated-session protocols in outpatient or community environments [18,91,93,94]. At the same time, measurement approaches were not always comprehensive; the Virtual Environment for Spatial Processing Assessment 2.0 feasibility work explicitly cautioned that relying on a single usability scale is insufficient to capture the multidimensional nature of feasibility, usability, and UX in complex VR rehabilitation systems [18]. This aligns with the broader need to report usability, tolerability, workload, and adverse events in parallel, particularly for populations at risk of fatigue, dizziness, or anxiety during immersion [93,99,100].

Qualitative and mixed methods evidence further clarifies what drives (or undermines) positive UX. Think-aloud evaluation of a VR module identified concrete interaction and interface-level issues that can influence comprehension, confidence, and task flow, underscoring the value of formative testing before efficacy trials [105]. Broader experiential accounts emphasized that perceived realism, meaningfulness of activities, and alignment with older adults’ routines shape engagement and perceived usefulness [92,103,104]. Cross-cultural comparison during an immersive VR cognitive task highlighted that UX is not solely determined by hardware or task difficulty, but also by contextual factors that may influence comfort and interpretation of the virtual environment [95]. Complementing self-report, head-kinematics–based modeling demonstrated that behavioral signals during VR gameplay can be leveraged to characterize UX and its associations with cognitive factors, suggesting a pathway toward more objective, continuous UX monitoring in future systems [98].

Several studies explicitly adopted human-centered or participatory design strategies, reinforcing that usability in older and cognitively impaired populations benefits from iterative refinement and stakeholder inclusion. Participatory design in dementia-related VR physical rehabilitation foregrounded feasibility in real-world care contexts and supported tailoring to users’ preferences and constraints [102]. In parallel, carers’ and professionals’ perspectives emphasized practical barriers and facilitators to uptake (eg, training requirements, perceived appropriateness, and care workflows), indicating that acceptance is not only an end user property but also an organizational and implementation outcome [101]. Iterative user-centered development of robot-mediated paired activities likewise illustrates how repeated testing cycles can be used to refine interaction patterns for older adults with MCI, particularly when social and motivational dynamics are central to the intervention experience [107].

Finally, the included set suggests an emerging trend toward adaptive and home-oriented solutions, while also revealing unresolved UX challenges. Combining reinforcement learning with VR for mild neurocognitive impairment reflects a move toward personalization and dynamic difficulty regulation, with usability assessment extending to both patients and caregivers [97]. Home-based or remotely oriented cognitive rehabilitation tools (including app-based platforms) further broaden access but amplify the importance of intuitive interaction, autonomy support, and long-term engagement over time [96,106,108]. Overall, the evidence indicates that VR or AR systems are frequently acceptable and usable in controlled pilots, but comparability is limited by heterogeneous UX constructs and reporting practices. Future trials should therefore adopt multidimensional usability frameworks, report stakeholder-specific acceptance (patients, carers, and clinicians), and integrate objective interaction markers alongside standardized questionnaires to better explain adoption and adherence [18,98,100,101].

### VR or AR for Diagnosis and Assessment (ADL or IADL, Spatial Navigation, and Functional Skills)

The studies in this subgroup leverage VR or AR (and closely related interactive 3D systems) primarily as measurement tools to assess clinically relevant cognitive-functional constructs with greater ecological validity than traditional clinic-based tests (Table 4). A clear concentration is observed in (1) spatial navigation and orientation as sensitive markers of age-related decline and MCI-related impairment, and (2) functional skills or ADL-IADL performance captured through structured, everyday-like scenarios. Across the subgroup, VR systems are used either as standalone diagnostic probes or as performance-based end points embedded within feasibility, validation, or intervention protocols, with heterogeneous outcomes and reporting practices limiting direct cross-study comparability.

**Table 4.** VR^a^ or AR^b^ for diagnosis and assessment (ADL^c^ or IADL^d^, spatial navigation, and functional skills).

Study (year)	Population or stage	Design and sample (N)	Technology and intervention	Dose and key cognitive outcomes
Mccracken et al (2025) [109]	Healthy younger adults vs healthy older adults; no diagnosed cognitive impairment	Cross-sectional experimental study; within-subject comparison of real vs VR homing; N≈40 (younger+older adults)	VR navigation task (HMD^e^) plus matched real-world corridor task—Fully immersive HMD VR Participants performed homing trials in both real and virtual replicas of an indoor environment; accuracy and path metrics compared across age groups and modal	Single laboratory session with multiple trials in each condition Domains: spatial navigation; path integration; visuospatial memory Outcome: older adults showed reduced homing accuracy in both environments; VR reproduced age-related navigation deficits observed in the real world.
Tuena et al (2024) [110]	Older adults with amnestic MCI^f^ and cognitively healthy controls	Experimental study with between-group comparison of navigation aids; N≈30-40 (MCI+controls)	Desktop VR maze or navigation task—nonimmersive desktop VR Participants learned routes in a virtual environment with different navigation aids (bodily vs visual-cognitive cues), followed by spatial recall tests	Single experimental session with several learning–recall blocks Domains: spatial memory; spatial navigation; executive functions Outcome: Bodily and visual-cognitive aids improved spatial recall, particularly in participants with MCI.
Silva et al (2023) [111]	Community-dwelling older adults without dementia	Feasibility and validation study; N≈40-50 older adults	Immersive VR outdoor-navigation scenario (SOIVET system)—fully immersive HMD VR; older adults explored a virtual neighborhood and performed egocentric orientation tasks (pointing or walking to remembered locations); usability and cybersickness	Single VR assessment session Domains: Egocentric spatial orientation; visuospatial memory; attention Outcome: Immersive VR assessment was feasible, well tolerated, and showed meaningful variability in egocentric orientation.
Tuena et al (2024) [112]	Older adults with MCI	Proof-of-concept interventional trial with VR training vs control; N≈30 MCI participants (training vs control)	Desktop VR navigation game with embodied interaction—nonimmersive or low-immersion VR Participants with MCI completed repeated embodied VR navigation tasks over several weeks; pre-post cognitive and navigation measures were collected	Multisession VR training over several weeks Domains: Spatial navigation; episodic memory; executive functions Outcome: VR-based embodied navigation training improved spatial navigation and some cognitive scores relative to controls.
Panerai et al (2023) [113]	Adults and older adults with major neurocognitive disorder (degenerative and nondegenerative etiologies)	Nonrandomized interventional study (VR training vs. control); N=67 (40 experimental; 27 control)	Tablet or PC-based nonimmersive VR applications simulating ADL or IADL tasks—nonimmersive VR Experimental group trained four functional living skills in virtual scenarios, with in vivo performance of the same tasks assessed at multiple time points	Structured multisession VR training with three assessment time points Domains: Functional living skills (ADL or IADL); executive functions; attention; memory Outcome: VR-trained participants improved both in virtual tasks and in real-world execution of the trained functional skills.
Stasolla et al (2024) [114]	People with Alzheimer disease and cognitively healthy controls (where applicable)	Comparative study of traditional vs VR-based or digital storytelling approaches; N=NR^g^ (to be extracted)	Nonimmersive VR or computer-based digital storytelling platform—nonimmersive VR or screen-based Patients engaged in digital storytelling activities delivered via VR-inspired interactive environments; outcomes compared with more standard assessment or rehabilitation	Multisession intervention over several weeks (details to be specified from full text) Domains: Episodic memory; autobiographical memory; executive functions; language Outcome: VR or digital-storytelling condition showed comparable or better gains in some cognitive and functional measures relative to standard approaches.
Mirmiran and Moussavi (2026) [115]	Institutionalized older adults with advanced dementia	Single-arm pilot intervention study; N=10 (9 completing ≥20 sessions)	Semi-immersive driving simulator with large display and steering controls—semi-immersive VR Participants repeatedly used a VR driving simulator presenting pleasant outdoor environments; quality of life and behavioral symptoms were monitored	Several sessions per week over approximately four months Domains: Well-being and engagement (primary); visuomotor coordination; secondary behavioral outcome Outcome: Feasible in advanced dementia; associated with improved quality-of-life measures and reduced behavioral disturbance in many residents.
Varela-Aldás et al (2022) [116]	Healthy adults (middle-aged and older)	Feasibility and validation study; N≈30 healthy participants	VR kitchen or cupboard scenario using an HMD—fully immersive HMD VR Participants memorized the location of everyday objects in virtual cupboards and later recalled or placed them; task metrics were related to standard neuropsychology	Single VR assessment session (~30-45 minutes) Domains: Everyday episodic memory; prospective memory; executive functions Outcome: Cupboard task was feasible, well accepted, and produced memory indices correlated with traditional tests, supporting ecological validity.
Bhargava et al (2025) [117]	Game developers and neuroscience/cognition researchers (no patient sample)	Qualitative mixed methods study (interviews, workshops); N=approximately 20-30 professionals	Digital serious games for cognition (including VR or 3D concepts)—mainly nonimmersive; conceptual discussion of multiple levels of immersion, explored design processes, constraints, and opportunities when cocreating games aimed at assessing cognition	Not applicable (design or consultation study) Domains: Multiple domains: attention, memory, executive functions, processing speed Outcome: Identified key design principles and tensions between playability and psychometric rigor for cognition-assessing games.
Mahncke (2025) [118]	Conceptual paper; focuses on clinical and research populations reporting cognitive complaints	Commentary or methodological article; N=not applicable	Not specific to one technology; implications for digital and VR-based assessments—not applicable, provides guidance on interpretation of self-reported cognitive complaints and their relation to objective performance measures	Not applicable Domains: Metacognition; subjective cognitive complaints; self-awareness of decline Outcome: Argues that self-report alone is insufficient as a diagnostic tool and should be combined with objective (including VR) assessments and longitudinal follow-up.

a VR: virtual reality.

b AR: augmented reality.

c ADL: activities of daily living.

d IADL: instrumental activities of daily living.

e HMD: head-mounted display.

f MCI: mild cognitive impairment.

g NR: not reported.

Several studies operationalize navigation using route learning, homing, and egocentric orientation tasks. A real-world versus virtual comparison of homing performance indicates that VR can reproduce age-related decrements in navigation or path integration, supporting the construct validity of VR-derived navigation metrics in older adults [109]. Immersive assessment of egocentric orientation in older adults further demonstrates feasibility of HMD-based protocols to quantify orientation performance while capturing usability- and tolerability-relevant factors inherent to immersive testing [111]. In MCI-focused work, desktop-based paradigms examine how navigation task design (eg, bodily and visual-cognitive aids) shapes spatial recall and diagnostic sensitivity, suggesting that the embedded cue structure may meaningfully influence performance and interpretability [110]. Related protocols extend to embodied spatial navigation paradigms where repeated task performance can be systematically tracked, offering a potential route to longitudinal assessment signatures (eg, learning curves, variability, and strategy shifts) rather than single-session snapshots [112].

A complementary line of research targets functional capacity via simulated everyday tasks (eg, shopping, medication management, and packing), thereby enabling standardized measurement of instrumental activities that are directly relevant to clinical staging and care needs [113]. Immersive everyday-memory assessment similarly embeds memory demands in familiar household contexts, providing object-location and prospective-memory–like indices intended to reflect real-world failures more directly than decontextualized neuropsychological tasks [116]. Collectively, these approaches illustrate how VR-based assessment can bridge the gap between laboratory cognition measures and functional outcomes, although the extent of transfer to real-world functioning remains variably demonstrated and often depends on task selection and scoring definitions.

In more advanced dementia, formal cognitive testing can be constrained by disease severity; therefore, studies sometimes prioritize pragmatic assessment outcomes, such as engagement, behavioral symptoms, and quality-of-life proxies within repeatable, structured experiences [115]. Other work uses VR-supported or VR-inspired digital storytelling frameworks to compare outcomes against more standard approaches, using the virtual environment as a scaffold for eliciting autobiographical or episodic content and monitoring response patterns [114]. In addition, immersive VR cognitive stimulation trials in AD incorporate performance-based and clinical outcome assessments as end points (even when the primary intent is therapeutic), providing data relevant to feasibility and evaluability of VR protocols in mild-to-moderate dementia [72].

In total, 2 contributions provide interpretive context for assessment-focused VR. A qualitative examination of collaboration between developers and neuroscience researchers highlights tensions between engagement-driven game mechanics and psychometric requirements, underscoring the importance of validity-by-design, telemetry planning, and transparency of scoring logic in cognition-assessing systems [117]. A methodological commentary on interpreting self-report cognition measures reinforces that subjective reports should not be used as standalone diagnostic indicators and motivates combining them with objective performance-based measures—including VR tasks—preferably within longitudinal frameworks [118]. Taken together, the subgroup supports VR or AR as a promising modality for assessing navigation, everyday memory, and functional capacity, but it also emphasizes the need for improved standardization (task definitions, scoring, adverse-event reporting, and benchmark validation) to strengthen synthesis and clinical interpretability across studies.

### Conceptual, Methodological, and Guideline Literature: Broader Context

Table 5 summarizes conceptual, methodological, and guideline-oriented publications that provide the broader context for VR- or AR-based approaches in cognitive rehabilitation and related functional outcomes in older adults. These papers do not primarily aim to estimate intervention efficacy; instead, they shape the field by (1) articulating design and usability guidance for older users, (2) proposing conceptual frameworks and taxonomies for intervention content and mechanisms, (3) outlining methodological pathways for development and evaluation, and (4) mapping research trends to identify gaps and priorities for scalable, reproducible work [2,14-16,21,30,119-126].

**Table 5.** Conceptual, methodological, and guideline literature: broader context.

Study (Year)	Population or stage	Design and sample (N)	Technology and intervention	Dose and key cognitive outcomes
Abeele et al (2021) [14]	Older adults (aged 55-95 y), diverse cognitive abilities or care settings; first IVR^a^ experience	Guideline paper: literature-derived guidelines+empirical qualitative study (interviews; laddering); N=37	Immersive VR^b^ (HMD^c^-based IVR); guidelines spanning content/hardware/context immersive (HMD IVR) compiled 67 design guidelines from literature; exposed 37 older adults to first IVR experience and interviewed them to empirically ground or refine guidelines	Design or accessibility or user experience focus; cognitive screening used in sample characterization, empirically grounded design guidance for accessible or engaging IVR for older adults; links IVR attributes to functional or psychosocial benefits
Seifert and Schlomann (2021) [15]	Older adults (focus on adults aged older than 75 y in Europe; heterogeneous abilities and technology experience)	Opinion or short communication (narrative overview)	VR and AR^d^ (broad use cases: health gaming, social contact, rehabilitation, and daily-life support) varies (not limited to a specific device class), discusses opportunities and barriers for older adults’ VR or AR use; proposes developer or research recommendations (participatory design, and training or support)	General cognition or ADL^e^ support context; highlights risks for users with cognitive limitations and cybersickness considerations, synthesizes key barriers (digital divide, skills or access, cybersickness, and environment constraints) and recommends participatory design and user support to improve adoption
Moon and Han (2022) [16]	People with neurological disorders (including cognitive impairment); accessibility for patients underexplored	Perspective paper	Immersive VR (stereo HMD) in neurology (rehabilitation+potential for diagnosis or assessment; metaverse context), immersive (explicitly defines VR as immersive stereo HMD), reviews current state of VR in neurology and proposes future directions, emphasizing VR-based cognitive assessment/diagnosis and accessibility or safety	Working memory, executive function, spatial navigation or spatial memory, cognitive planning (as examples for VR-based daily-life tasks), argues VR is underused for diagnosis or evaluation; proposes VR tasks simulating daily life with richer performance parameters and calls for standardization and diverse normative datasets
He et al (2022) [30]	Publications on VR-based cognitive rehabilitation (2001-2021); diseases include stroke, dementia, MCI^f^, etc	Bibliometric analysis (Web of Science; visualization or clustering tools); N=1259 papers	VR for cognitive rehabilitation (field-level mapping) varies (depends on included studies), retrieved WoS^g^ records (2001-2021) and analyzed authors, countries, keywords, and trends	Field-level: cognition or cognitive rehabilitation broadly maps research hotspots and trends; highlights telerehabilitation growth and notes lack of consensus on treatment methods and safety concerns (per authors)
Manser and de Bruin (2021) [120]	Older adults with mild neurocognitive disorder; project “Brain-IT” (design/development focus)	Methodological paper+case study applying Motivation–Interaction–Design–Evaluation framework	Exergame-based motor-cognitive training system (Dividat Senso or Senso Flex); gamification and multisensory feedback primarily nonimmersive exergaming (no HMD emphasized), describes step-by-step design or development process guided by MIDE (contextual research, game design or development, evaluation) to create exergame training aimed at halting or reducing	Motor-cognitive training (multiple domains; tailored to user capabilities and therapeutic needs) provides replicable methodological workflow and design requirements to increase usability, acceptance, and adherence of exergame interventions in clinical practice
Guzmán et al (2024) [21]	Older adults (cognitive rehabilitation context; framework-level; expert review included clinicians and therapists)	Conceptual framework development (Jabareen methodology)+expert review; N=5 experts (framework review)	Serious games for cognitive rehabilitation; emphasizes VR; includes AR as proposed extension primarily immersive VR context (mentions HMD; also discusses AR integration), develops a conceptual framework linking VR, physical activity, social interaction, difficulty adaptation, and content customization	Multiple domains (framework emphasizes multidomain cognitive rehab+real-world scenarios; examples include executive functions and others), Framework intended to guide design and implementation of serious games for older adults’ cognitive rehabilitation; highlights iterative, multidisciplinary, participatory development and personalization
Cheng et al (2025) [119]	Older adults (community-dwelling); cognitive and independent living skills training in a home environment simulation	Feasibility study (qualitative interviews after gameplay); N=30	VR serious game (“Brainland”) in home-like virtual environment with mini-games Immersive VR (HMD implied) designed a VR game with 6 mini-games targeting learning or cognitive and independent living skills; evaluated feasibility via semistructured interviews after play	Single trial session ~20 minutes (per author), cognitive skills+independent living skills (life skills or ADLs); mentions attention, working memory, processing speed as longer-term targets: Positive perceived usefulness of use and reported competency or cognitive developments; identifies engagement benefits and negative experiences (cybersickness or anxiety) informing best practices

a IVR: immersive virtual reality.

b VR: virtual reality.

c HMD: head-mounted display.

d AR: augmented reality.

e ADL: activities of daily living.

f MCI: mild cognitive impairment.

g WoS: Web of Science.

Empirically grounded guidance underscores that acceptability and UX are not peripheral outcomes but prerequisites for any clinically meaningful deployment. Design recommendations for immersive VR tailored to older adults emphasize onboarding, interaction simplicity, comfort, and accessibility as core requirements for both clinical and home use [14]. Broader discussions of VR or AR adoption in later life further highlight structural and individual barriers (eg, technology access, digital skills, perceived relevance, and tolerability), motivating usability-by-design and inclusive implementation strategies [15]. Additional works in this group complement these contributions by further operationalizing usability, safety, and user-centered evaluation considerations relevant to older populations [124,126].

Conceptual papers provide shared vocabularies for describing intervention components (eg, task structure, feedback, engagement mechanisms, and functional relevance), which is essential for comparing heterogeneous VR or AR studies within systematic reviews. A conceptual framework for serious games for cognitive rehabilitation synthesizes design and content considerations intended to support both therapeutic intent and long-term engagement [21]. Related conceptual and synthesis contributions in this set further refine how VR or AR interventions can be specified and categorized, supporting clearer reporting and improved cross-study comparability [122,123].

Methodological publications stress iterative, user-centered development and transparent reporting of technology characteristics (hardware, interaction modality, and immersion level) and intervention parameters (dose, progression, and feedback). A structured methodological approach to exergame development illustrates how therapeutic goals can be aligned with game mechanics and feasibility constraints in older adults with neurocognitive disorders [120]. Complementary methodological contributions in this group further strengthen evaluation pipelines (eg, feasibility-to-trial progression, outcome selection, and implementation-relevant metrics) to reduce variability and improve reproducibility [2,125].

Perspective and bibliometric studies contextualize why certain paradigms, outcomes, and technologies dominate the literature and where underexplored opportunities remain. A neurological disorder perspective highlights both the promise of immersive VR for ecologically valid task simulation and measurement and the practical considerations affecting translation [16]. Bibliometric mapping of VR in cognitive rehabilitation characterizes publication growth and thematic concentrations, informing priorities, such as methodological standardization, reporting quality, and scalability [30,121]. Additional mapping work (including domain-wide bibliometric analyses) and interaction-technology–focused syntheses (eg, hand-tracking interfaces used in VR-based assessment or intervention) further contextualize research growth and methodological gaps relevant to scalable deployment and measurement fidelity [41,127].

Finally, feasibility-oriented design work demonstrates how the above principles can translate into ecologically meaningful content and UX evidence in older adults, providing practical signals on tolerability and perceived usefulness that can guide refinement and future trial design [119].

Overall, this body of context literature supports three interpretive lenses for the present evidence synthesis, that are (1) usability and acceptability should be treated as core outcomes rather than secondary considerations [14,15,124,126], (2) heterogeneity in study designs and outcomes can be partly mitigated through clearer specification of intervention components and technology characteristics [2,21,122,123,125], and (3) future research should prioritize methodological rigor, standardized reporting, and scalable deployment pathways to move beyond proof-of-concept demonstrations [16,30,119,121].

## Discussion

### Principal Findings

This systematic review synthesizes evidence on immersive technologies for cognitive rehabilitation across the MCI-dementia continuum, with an explicit focus on usability, therapeutic efficacy, and limitations spanning VR, AR, and CAVE-like embodied systems. Overall, the literature indicates that immersive interventions are feasible and frequently engaging, with signals of benefit across multiple cognitive domains (eg, attention, memory, executive functions, and spatial navigation), but the strength of conclusions remains constrained by substantial heterogeneity in technology, protocols, and outcomes, as well as the predominance of small-sample and early-phase designs [2,4,20,23-26].

**Figure 3.**
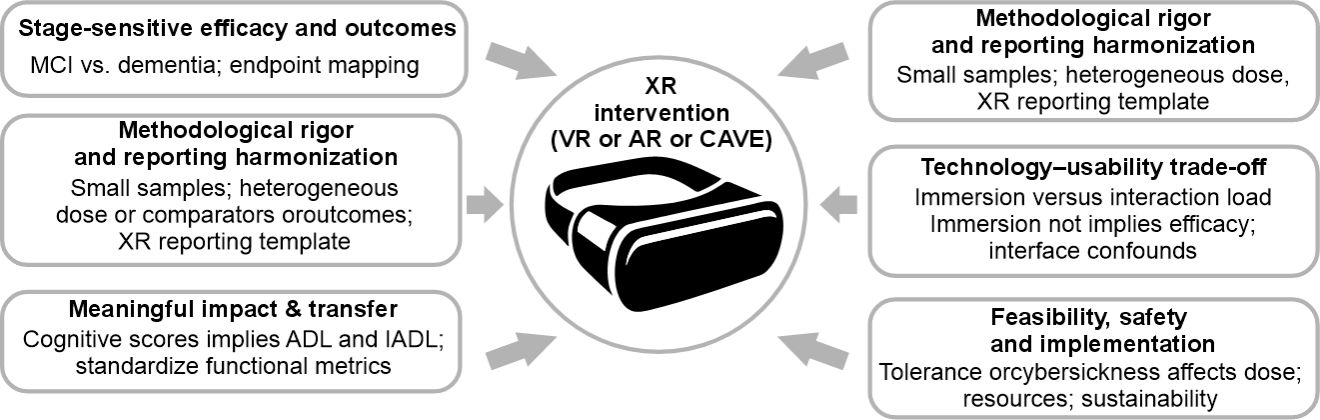
Key challenges for immersive extended reality cognitive rehabilitation (VR, AR, or CAVE) across the mild cognitive impairment–dementia continuum: stage-sensitive outcomes, immersion–usability trade-offs, functional transfer (ADL or IADL), methodological or reporting harmonization, and feasibility, safety, and implementation at scale. ADL: activities of daily living; AR: augmented reality; CAVE: Cave Automatic Virtual Environment; IADL: instrumental activities of daily living; MCI: mild cognitive impairment; VR: virtual reality; XR: extended reality.

Figure 3 provides a schematic overview of the core challenges shaping the interpretation, synthesis, and real-world applicability of immersive XR-based cognitive rehabilitation across the MCI-dementia continuum. The diagram positions the XR intervention at the center and highlights 5 interrelated domains that structure the subsequent discussion. First, therapeutic goals and outcome selection must be stage-sensitive, reflecting fundamental differences between restorative aims in MCI and maintenance-oriented or quality-of-life outcomes in dementia. Second, the figure emphasizes the trade-off between technological immersion and usability, illustrating how interaction demands and interface complexity can confound cognitive outcomes. Third, it underscores the persistent gap between improvements on cognitive test scores and meaningful functional transfer to ADL or IADL. Fourth, methodological rigor and harmonized reporting are identified as prerequisites for cumulative evidence synthesis in a field characterized by heterogeneous protocols and outcome measures. Finally, feasibility, safety, and scalability are highlighted as cross-cutting determinants of effective dose, adherence, and implementation beyond controlled laboratory settings. Together, these interconnected dimensions frame the interpretation of the current evidence base and guide priorities for future research and clinical translation.

### Interpretation of the Evidence Base Across MCI and Dementia

Across MCI, immersive training is commonly positioned as a restorative or capacity-enhancing approach, aiming to improve domain-specific performance (eg, executive control, working memory, and visuospatial abilities) and to strengthen skills relevant to everyday functioning [4,13,20]. In dementia, intervention goals often shift toward maintaining function, supporting compensatory strategies, and enhancing quality of life rather than achieving large gains on conventional neuropsychological end points; this distinction has important implications for outcome selection, intervention dose, and expectations of change [5,6]. Reviews spanning dementia or AD-oriented rehabilitation emphasize the value of ecologically meaningful tasks and the importance of aligning training scenarios with the functional challenges faced by patients and caregivers [5,6,17]. Taken together, the field would benefit from more explicit stage-sensitive theories of change—what should improve (or be preserved), for whom, and through which mechanisms in MCI versus dementia [16,21].

### Technology Matters: Immersion, Interaction Demands, and Ecological Validity

A core promise of immersive systems is ecological validity, the ability to engage cognitive processes within realistic, context-rich activities (eg, navigation, shopping-like scenarios, and multistep functional routines) that may better approximate everyday demands than traditional desktop-based training [13,17,112]. However, greater immersion is not synonymous with better outcomes. Higher immersion can increase interaction complexity and sensory load, which may introduce usability barriers for older adults with cognitive, motor, or sensory constraints and can confound interpretation (eg, performance differences driven by interface demands rather than the targeted cognitive construct) [8,14]. Accordingly, the usability literature emphasizes that interaction design, onboarding, and accessibility features are prerequisites for meaningful interpretation of outcome data, particularly in populations susceptible to fatigue, confusion, or anxiety during first exposure to novel technology [14,15].

AR remains less represented than VR in the cognitive rehabilitation literature, but it is conceptually attractive for supporting in situ cueing, errorless learning, and context-aware scaffolding of everyday activities [8,15,67]. A key research priority is to clarify where AR may offer a comparative advantage (eg, functional cueing and transfer) versus where VR may be preferable (eg, controlled manipulation of stimuli and graded exposure), while using comparable outcome frameworks that allow cross-modality synthesis [21,122].

### From Cognitive Test Scores to Meaningful Functional Outcomes

Many studies and syntheses emphasize cognitive test performance; yet, fewer consistently demonstrate robust transfer to ADL or IADL, participation, or caregiver-relevant outcomes [4,17,20]. This gap is particularly consequential for dementia, where functional preservation and symptom management may be primary goals [5,6]. XR platforms are well-suited to embed functional tasks and to collect rich, process-level behavioral data (eg, error patterns, sequencing, and wayfinding efficiency), but these measures are rarely standardized across studies, limiting comparability and interpretability [13,124,126]. Future trials should prespecify clinically meaningful primary end points that map to the target population and intervention rationale and should report functional measures with sufficient detail to enable aggregation and replication [21,122,123].

### Usability, Tolerability, and Safety as Determinants of Feasibility and Interpretability

Usability and acceptability are not secondary considerations in this domain; they are prerequisites for feasibility and for interpretable outcome measurement. Design and evaluation guidance for older users emphasizes minimizing interaction complexity, providing clear instructions and feedback, and ensuring comfort and accessibility in immersive experiences [8,14]. Cybersickness and related adverse effects can further influence adherence and exposure (eg, selective withdrawal, shortened sessions, and reduced “dose”), complicating cross-study comparisons and the interpretation of reported changes. Evidence focused on fully immersive VR in older adults indicates that cybersickness is a material barrier that warrants systematic monitoring and reporting, alongside mitigation strategies, such as gradual acclimation, careful locomotion design, and appropriate session duration [50]. More consistent reporting of adverse events, tolerability, and reasons for withdrawal is therefore essential for evaluating real-world feasibility and for understanding which systems and protocols are most likely to translate beyond controlled settings [14,15].

### Methodological Limitations and Sources of Heterogeneity

The current evidence base is constrained by several recurring methodological issues, such as (1) small sample sizes and early-phase designs, (2) variability in comparator conditions and control interventions, (3) wide variation in dose and supervision, and (4) heterogeneous outcome measures and reporting practices [2,4,20,23]. These limitations reduce statistical power and complicate meta-analytic synthesis, while also increasing the risk that positive findings reflect context-specific effects rather than generalizable efficacy. Methodological and reporting frameworks emphasize the importance of clear intervention specification, stage-appropriate evaluation (feasibility vs efficacy), and transparent reporting of implementation conditions (training, supervision, equipment, and setting) [119,120,122,123]. The field would benefit from harmonized reporting templates tailored to XR rehabilitation (technology characteristics, immersion level, interaction modality, content design, progression rules, and support requirements), enabling cumulative synthesis across platforms and sites [16,21,122].

### Scalability and Implementation in Real-World Care Pathways

Beyond efficacy, the practical deployment of XR interventions depends on scalability constraints, such as costs, maintenance, space requirements (particularly for room-scale systems), clinician time, and the need for caregiver involvement. Field-level syntheses and trend analyses consistently identify implementation, standardization, and scalability as major barriers to broader clinical uptake [16,30,121]. For home or community delivery, studies should evaluate feasibility under realistic constraints (digital literacy, connectivity, supervision, and safety) and should explicitly report the resources required for long-term use (setup time, troubleshooting, and staff training) [15,119]. In parallel, implementation of science constructs (eg, acceptability, feasibility, fidelity, and sustainability) should be incorporated prospectively rather than inferred post hoc [14,120].

### Emerging Opportunities: Personalization, Multimodal Sensing, and Mechanistic Evaluation

Several contemporary directions may increase the clinical relevance of immersive rehabilitation. First, adaptive personalization (rule-based or data-driven) may better accommodate heterogeneity in baseline impairment, fatigue, and learning profiles across MCI and dementia, but it requires transparent specification and validation to avoid “black-box” intervention effects [21,122]. Second, integration with multimodal sensing (eg, physiological measures and behavioral telemetry) may enable more sensitive monitoring of cognitive load, engagement, and progression but raises methodological questions about reliability, privacy, and clinical interpretability [3,125]. Finally, stronger mechanistic evaluation—linking task demands to hypothesized cognitive processes and to functionally meaningful outcomes—is needed to move from promising prototypes to reproducible treatment programs [13,16,21].

### Strengths and Limitations of This Review

A key strength of this review is the integrated synthesis of usability, therapeutic efficacy, and limitations across immersive modalities, rather than treating usability as a secondary concern or focusing narrowly on 1 technology class [8,14]. Nevertheless, our conclusions are bounded by the limitations of the underlying literature, including heterogeneity in intervention content and outcomes, and the frequent reliance on pilot studies. As in many rapidly evolving technology domains, publication and selective-reporting biases remain plausible, and inconsistent reporting of implementation factors limits external validity judgments [4,16,122].

### Implications for Clinical Practice and Future Research

From a practice perspective, the evidence supports cautious optimism—immersive interventions appear feasible and engaging for many individuals with MCI and, under appropriate support conditions, for some people living with dementia [5,6,14]. However, adoption should be guided by usability screening, conservative session dosing, and systematic monitoring of adverse effects, particularly in fully immersive VR [14,50].

For research, priorities include (1) standardized reporting of technology and intervention components, (2) stage-sensitive outcome frameworks linking cognition to ADL or IADL and quality of life, (3) adequately powered comparative trials with transparent comparator selection, (4) routine usability and safety reporting, and (5) implementation-focused evaluation to support scalability [16,21,30,119-123]. Addressing these challenges will improve the cumulative value of future studies and help transform immersive cognitive rehabilitation from a promising research area into a dependable component of care across the MCI-dementia continuum.

### Conclusion

This systematic review synthesized current evidence on immersive technologies (VR, AR, and CAVE) for cognitive rehabilitation in MCI and dementia, with an emphasis on therapeutic outcomes, usability, and translational limitations. Overall, the literature suggests that immersive interventions are feasible and often engaging, with promising signals of benefit across multiple cognitive domains. However, the strength and generalizability of conclusions remain limited by substantial heterogeneity in intervention content, technology and immersion levels, interaction modalities, dosage, and outcome selection, alongside the frequent use of small samples and early-phase study designs.

A key implication is that outcome interpretation cannot be separated from usability and tolerability. In cognitively impaired older adults, interface complexity, onboarding requirements, comfort, and adverse effects can influence adherence and delivered exposure (ie, the effective “dose”), thereby shaping observed performance and functional outcomes. Future studies should therefore treat usability, safety, and acceptability as core outcomes, report them transparently (including adverse events and reasons for withdrawal), and analyze them alongside cognitive and functional measures.

For the next generation of research, priorities include (1) clearer specification and reporting of intervention components (tasks, feedback, progression, and supervision), (2) better alignment of outcomes with clinically meaningful goals, including functional end points (ADL or IADL) and quality-of-life measures, (3) adequately powered comparative trials with well-justified control conditions, and (4) explicit evaluation of implementation and scalability, particularly for home and community deployment. Advancing methodological standardization and reporting consistency will be essential to enable cumulative synthesis and to support translation of immersive cognitive rehabilitation into dependable, real-world care pathways across the MCI-dementia continuum.

## Supplementary material

10.2196/84349 Checklist 1 PRISMA checklist.

## Abbreviations

AD: Alzheimer disease
ADL: activity of daily living
AR: augmented reality
CAVE: Cave Automatic Virtual Environment
HMD: head-mounted display
IADL: instrumental activity of daily living
MCI: mild cognitive impairment
MMSE: Mini-Mental State Examination
MoCA: Montreal Cognitive Assessment
PRISMA: Preferred Reporting Items for Systematic Reviews and Meta-Analyses
UX: user experience
VR: virtual reality
WoS: Web of Science
XR: extended reality
